# Do agricultural grasses bred for improved root systems provide resilience to machinery‐derived soil compaction?

**DOI:** 10.1002/fes3.227

**Published:** 2020-07-05

**Authors:** Nuwan P.K. Muhandiram, Mike W. Humphreys, Rhun Fychan, John W. Davies, Ruth Sanderson, Christina L. Marley

**Affiliations:** ^1^ Institute of Biological, Environmental and Rural Sciences (IBERS) Aberystwyth University Wales UK

**Keywords:** *Festulolium*, forage yield, roots, ryegrass, soil compaction

## Abstract

The increasing frequency of droughts and floods on grasslands, due to climate change, increases the risk of soil compaction. Soil compaction affects both soil and forage productivity. Differing grasses may counteract some effects of compaction due to differences in their root architecture and ontogeny. To compare their resilience to soil compaction, three *Festulolium* (ryegrass and fescue species’ hybrids) forage grass cultivars comprising differing root architecture and ontogeny were compared in replicated field plots, together with a ryegrass and tall fescue variety as controls. Pre‐compaction soil and forage properties were determined in spring using > four‐year‐old plots to generate baseline data. Half of each field plot was then artificially compacted using farm machinery. Forage dry matter yield (DMY) was determined over four cuts. After the final harvest, post compaction soil characteristics and root biomass (RB) were compared between grasses in the non‐compacted and compacted soils. Pre‐compaction data showed that soil under *Festulolium* and ryegrass had similar water infiltration rates, higher than soil under tall fescue plots. Tiller density of the *Festulolium* at this time was significantly higher than fescue but not the ryegrass control. Forage DMY was significantly lower (*p* < .001) with compacted soil at the first cut but, by the completion of the growing season, there was no effect of soil compaction on total DMY. Tall fescue had a higher total DMY than other grasses, which all produced similar annual yields. Soil bulk density and penetration resistance were higher, and grass tiller density was lower in compacted soils. Root biomass in compacted soils showed a tendency for *Festulolium* cv Lp × Fg to have higher RB than the ryegrass at 0–15 cm depth. Overall, findings showed alternative grass root structures provide differing resilience to machinery compaction, and root biomass production can be encouraged without negative impacts on forage productivity.

## Graphical abstract

The increasing frequency of droughts and floods on grasslands, due to climate change, increases the risk of soil compaction, affecting both soil, and forage productivity. Differing grasses may be able to counteract some of these effects of compaction due to differences in their root architecture and ontogeny. To compare their resilience to soil compaction, three Festulolium (ryegrass and fescue species’ hybrids) forage grass cultivars comprising differing root architecture and ontogeny were compared in replicated field plots, together with a ryegrass and tall fescue variety as controls.

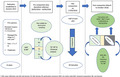

## 1 INTRODUCTION

Livestock farming is a significant component of the total UK agricultural output, calculated to be worth £14.4b in 2017, 1.5 times higher than all other UK crops combined (Defra, 2017). However, despite their importance to the UK economy, various pressures, both political and environmental, threaten the future sustainability of livestock farming both in the UK and elsewhere (FAO, 2018).

Diverse weather extremes are becoming increasingly common in the UK. Overall trends indicate a UK climate with drier summers and wetter winters than experienced in previous years (Meteorological Office, 2018). Encounters of contrasting rainfall extremes may exacerbate threats to grassland perpetuity. Incidents of extreme rainfall result in increased risks of surface water runoff from grasslands, especially on slopes and in shallow soils, or from previously water‐logged soils. The likelihood of surface runoff will be exacerbated where soils have shrunken and dried following previous exposure to prolonged drought conditions. Under both flood or water conditions, grass growth may be reduced and its persistency compromised. The impacts of droughts on perennial ryegrass (*Lolium perenne L*.) and in particular Italian ryegrass (*Lolium multiflorum* Lam.) have been demonstrated (Aper, Ghesquiere, Cougnon, & Baert, 2014; Humphreys, Pasakinskiene, James, & Thomas, 1998; Humphreys et al., 2006). Intensive rains and flooding will deter grassland establishment, and later in the season, will incur delays in harvesting which will reduce forage quality due to increased grassland maturity (Cherney & Hall, 2008). In addition to the negative impacts on grass crop production, detrimental effects on soil structure, function, and biota composition, and on soil carbon influx, may also occur (Karmakar, Das, Dutta, & Rakshit, 2016). At high soil moisture and low organic matter, grassland soils are more vulnerable to compaction, caused mainly by animal traffic and farm machinery movements (Hamza & Anderson, 2005).

Soil compaction has been identified as a major problem in modern agricultural grasslands. AHDB (2015) found that as much as 70% of grassland soils in England and Wales showed signs of compaction as a consequence of damage from livestock and/or farm machinery, highlighting the need for further research. Grasses with extensive root systems may improve soil qualities through soil–root interactions (Humphreys, O'Donovan, Farrell, Gay, & Kingston‐Smith, 2014; Kell, 2011; Marshall, Collins, Humphreys, & Scullion, 2016). Advances in new root screening technologies have provided opportunities for the incorporation of selections for root growth and design in plant breeding strategies. The differences in root architecture and changes in the ontogeny of certain *Festulolium* cultivars through the growing season have been described elsewhere and compared to currently used perennial ryegrass varieties during equivalent timelines (Macleod et al., 2013 and Humphreys et al., 2018). Humphreys et al. (2018) found that certain *Festulolium* hybrids had more extensive root systems, particularly at depth, when compared to *Lolium perenne* L. (perennial ryegrass). Such deeper root systems should improve water (Durand et al., 2007) acquisition and aide soil hydrology (MacLeod et al., 2013) and carbon sequestration at depth in soils (Kell, 2011).

One of the current global challenges facing agriculture is the greater need for grassland that can deliver both food security and environmental benefits in order to provide farmers with the tools they need to help combat the impacts of climate change on food production. In the current study, three *Festulolium* grass cultivars of different genome composition were compared against each other and against perennial ryegrass and tall fescue varieties used in current grassland agriculture. The aim was to evaluate the impact of soil compaction on the above‐ and below‐ground agronomic performance of the grasses to determine whether these novel grasses can provide the same productivity of forages while delivering environmental benefits in terms of grass–soil interactions.

## 2 MATERIALS AND METHODS

### 2.1 Experimental site and treatments

An experiment was conducted at the Institute of Biological, Environmental and Rural Sciences (IBERS), Wales, Aberystwyth (52° 26' 5" N, 4° 0' 28" W, 40 m of altitude) on stony, well‐drained loam soil of the Rheidol series over a nine‐month period (February 2016 – October 2016) (site rainfall, air, and soil temperatures are given in Table S1). Three replicated 5 × 1.5 m experimental grass plots arranged in randomized blocks had previously been established in autumn 2012. Prior to the current research, plots were maintained under a five cut year^‐1^ regime to simulate conservation management for silage using conventional NIAB field management protocols (Humphreys et al., 2014). In the current study, similar protocols for the management of the field plots were continued, but only four grass cuts were taken over the growing season between spring and autumn in 2016 due to a cold spring which initially delayed plant growth. Fertilizer was applied in two applications prior to the first cut, in early March and in early April, and immediately following cuts 1–3 providing a total of 356 kg N, 62 kg P_2_O_5_, 201 kg K_2_O, and 108 kg SO_3_ ha^‐1^ annum^‐1^.

The grasses evaluated comprised three tetraploid *Festulolium* cultivars: (i) *Lolium perenne* L. × *Festuca mairei* Hack. (Lp × Fm); (ii) *Lolium perenne* L. × *Festuca arundinacea var*.* glaucescens* Roth. (Lp × Fg); and (iii) *Lolium perenne* L. × *Festuca pratensis* Huds. cv Prior (Lp × Fp). The forage yield and root ontogeny of the three *Festulolium* cultivars in comparison to ryegrass have been reported previously (Humphreys et al., 2014, 2018; Macleod et al., 2013). In the current experiment, Lp × Fm, Lp × Fg, and Lp × Fp were compared to two grass varieties, used currently in livestock agriculture, selected as controls based on their high field performance and also their genetic relationship to the three *Festulolium* cultivars. They were perennial ryegrass (*Lolium perenne* L.) cv. AberBite (4x) and Tall fescue (*Festuca arundinacea* Schreb.) cv. Kora (6x). In all field plots, the grasses used were of equivalent development, persistency, and ground cover with no bare soil visible.

For the compaction treatment, randomly selected 1.5 × 2.1 m halves of each grass subplot were compacted (17 March 2016) using a 2,040 kg weight Cambridge ring roller applied over six tractor passes plot^‐1^. The efficacy of the method used to compact soil was demonstrated previously by Glab (2013) who reported increased penetration resistance (PR) in 0–20 cm soil layer given the same soil compaction treatment. The procedures applied in the current study ensured the compaction of soil structures and provided the opportunity to compare soil characteristics and below and above ground growth of the five grasses under non‐compacted (NC) and compacted (C) soil conditions (SC).

### 2.2 Measurements

Both soil and forage characteristics were determined at two time points: a) prior to compaction (baseline) and b) post compaction.

#### 2.2.1 Soil measurements

##### Prior to compaction

Pre‐compaction soil data were obtained between 26 February and 16 March 2016. Measurements on soil physical and chemical properties and on grass development above ground were collected. The 1.5 × 5 m plots were subdivided into three sections comprising two 1.5 × 2.1 m areas either side of a median subsection of 1.5 × 0.8 m. The median subsection was used for destructive soil sampling (viz. water infiltration rate, bulk density in order to minimize the impact of these activities on soil and forage yield measurements taken in the remaining areas of each field plot and to ensure no carryover effect, for example run off between 1.5 × 2.1 m subplots.

Water infiltration rate (WIR) and penetration resistance (PR) were measured in situ with the vegetation present and soil samples were collected to examine bulk density (BD) and soil chemical composition. Water infiltration rates were determined using double ring infiltrometers (ASTM, 2003; Bodhinayake, Si, & Noborio, 2004) after three consecutive dry days following an episode of heavy rain. A Field Scout SC 900 soil compaction meter (Spectrum Technologies, Inc. Aurora) with a 12 mm cone was inserted to a minimum of 35 cm depth at constant insertion rate to assess soil PR. The PR profiles taken in randomized positions (12 plot^‐1^) were collated to a depth of 30 cm at 2.5 cm intervals. Bulk density was determined using three soil cores of 5.7 cm diameter × 5.9 cm deep. For soil N, pH, and mineral analysis, soil samples were collected using a soil corer at 0 – 7.5 cm depth in a “W”‐formation across each plot. Twelve soil cores were bulked for each plot. Soil N was measured as nitrate (NO_3_^‐^‐N) and ammonium‐*N* (NH_4_^+^‐N). Fresh soil samples were sieved through a 12‐mm mesh, and subsamples were taken immediately to determine dry matter (DM), NH_4_^+^‐N_,_ and NO_3_^‐^‐N content. Soil DM was determined by drying 100 g samples (one representative sample plot^‐1^) at 105°C for 48 hr. For the determination of NH_4_^+^‐N and NO_3_^‐^‐N, fresh soil samples (10 g) were shaken with 2M potassium chloride (50 ml) solution for 1h and then filtered. Nitrate nitrogen was determined by reduction of nitrate to nitrite using a cadmium column followed by colorimetric measurement at 520nm while NH_4_^+^‐N was determined at 660 nm. Soil pH, P, K, Ca, and Mg were analyzed as described in Crotty et al. (2014).

##### Post compaction

WIR, BD, and PR were measured between 4 October and 24 November 2016, using the same methods described above for the pre‐compaction observations.

#### 2.2.2 Forage measurements

Tiller density (TD) was used as an indicator of grass growth and resilience to compaction and for rooting potential since the base of each grass tiller is a potential root source (Matthew, Van Loo, Thom, Dawson, & Care, 2001). Prior to soil compaction, grass tiller numbers within a 36 × 25 cm quadrat were measured at three randomly selected locations in each 1.5 m × 5 m plot. After the final forage cut, post compaction tiller densities were determined from three quadrats within each 1.5 m × 2.1 m subplot.

Root biomass (RB) was determined in soil cores collected on 24 November 2016 from non‐compacted and compacted subplots using an 8 × 15 cm diameter root auger (Eijkelkamp, Giesbeek, Netherlands). The auger measurements were taken from central plot locations deemed a sufficient distance from each plots’ edge to negate any potential “edge effects.” A soil core was extracted from each subplot in two sections 0–15 cm and 15–30 cm deep. Soil cores were stored at 4°C for a maximum of 7 days prior to processing. Following the removal of foliage at the soil surface within the upper soil core, all soil cores were immersed in cold water for approximately 45 min. Roots were carefully separated manually from the thoroughly soaked soil cores by pouring through a stack of three sieves of mesh sizes 2.8 mm, 710, and 600 µm. All small roots that passed through the 2.8 mm mesh were collected together with fine soil particles and any other debris. These were rinsed further using pressure pumped water with care taken not to lose any of the roots. Each sieve was oscillated gently in a clean water bath in order to extract small stones or soil particles at the bottom, while most of organic debris could be separated by flotation. All washed roots (new and old) were collected manually using hand forceps, and any remaining soil debris was removed. Root dry weight in each 7.5 cm core was determined following drying at 80°C for 48 hr.

Four grass cuts were harvested (Cut 1:17 May 2016, Cut 2:27 June 2016, Cut 3:15 August 2016, and Cut 4:4 October 2016) from both non‐compacted and compacted field subplots using a Haldrup 1500 plot harvester (J. Haldrup a/s, Løgstør, Denmark) at a cutting height of 5 cm, in accordance with standardized IBERS protocols to simulate a silage cutting height. At each harvest, fresh weight yield was determined immediately after cutting and harvested forage was sub‐sampled for DM determination and assessment of botanical composition. Forage DM was determined by oven drying samples at 100°C for 24 hr. Botanical composition was determined as described by Marley, Fychan, Fraser, Sanderson, and Jones (2007). In brief, forage samples were divided into sown grass, weed grasses, and dicotyledonous broad‐leaf weed species.

### 2.3 Statistical analysis

Pre‐compaction soil and forage‐based data were analyzed by analysis of variance (ANOVA) of the randomized complete block design with the grass cultivar used as the treatment factor and using GenStat^®^ (Release 19; Baird, Murray, Payne, & Soutar, 2017). Post compaction data were analyzed by ANOVA of the 5 × 2 factorial according to the split plot design with effects of grass treatments estimated at the plot level and effects of compaction and its interaction with grass estimated at the subplot level. Data transformations were applied where necessary to normalize the data prior to analysis.

Total soil penetration resistance between 0 and 30 cm soil depth was calculated as the area beneath the profile for PR versus depth. Treatment effects on the profiles post compaction were examined by repeated measures ANOVA. Effects of compaction at each depth were tested for statistical significance using *t* tests with the comparison‐wise type I error rate (*α*) adjusted using the Bonferroni method (i.e., *α*/total number of pairwise comparisons). The Student–Newman–Keuls method was used for multiple comparisons of means when there was a significant effect of grass. Where a significant grass × soil compaction interaction was found grass means within soil compaction treatment and soil compaction treatment, means within grass species were compared using *t* tests, adjusted as detailed for penetration resistance data.

## 3 RESULTS

### 3.1 Prior to soil compaction

Prior to soil compaction, the overall mean soil pH was 6.12 (*SEM* ± 0.04). All other soil physical variables and chemical composition data prior to soil compaction are presented in Table 1. There were no significant differences (*p* > .05) among the grass treatments in terms of soil bulk density nor soil chemical composition (*p* > .05). Penetration resistance through the uppermost 30 cm of soil was lower (*p* < .05) with the perennial ryegrass control when compared to two of the *Festulolium* cultivars, Lp × Fm and Lp × Fg, with Lp × Fp and tall fescue intermediate. Water infiltration rates were higher in soils underneath all three *Festuloliums* and ryegrass than under the tall fescue control (*p* < .05) (Table 1). In spring, prior to soil compaction, plant TD (no. of tillers 0.1m^‐2^) of all three *Festulolium* cultivars were higher than the tall fescue (*p* < .05), but did not differ significantly from the ryegrass control (Table 1).

**Table 1:** Pre‐compaction soil water infiltration rate (WIR), bulk density (BD), total penetration resistance 0 – 30 cm (PR), chemical properties and plant tiller density (TD) for three *Festulolium* cultivars (Lp × Fm (*Lolium perenne* L. (Lp) × *Festuca mairei* Hack. (Fm)), Lp × Fg (*Festuca glaucescens* Roth. (Fg)), Lp × Fp (*Festuca pratensis* Huds. (Fp)), and perennial ryegrass (Lp cv. AberBite) and tall fescue (*Festuca arundinacea* Schreb. cv. Kora) in spring 2016

	Ryegrass	Fescue	Lp × Fm	Lp × Fg	Lp × Fp	*SEM*	*P*‐value
Soil							
Bulk density (g DM cm^−3^)	1.32	1.30	1.32	1.29	1.34	0.030	.759
PR (kPa)	40370^a^	44805^a^	46530^b^	46958^b^	42908^b^	1,381.5	.049
WIR (cm/h)	0.30^b^	0.18^a^	0.50^b^	0.68^b^	0.40^b^	0.310^a^	.007
							
NO_3_^‐^‐*N* (mg/kg DM)	6.31	1.95	6.45	6.30	5.17	0.265^b^	.067
NH_4_^+^‐*N* (mg/kg DM)	4.53	3.75	6.70	5.80	3.65	0.070^c^	.107
P (mg/kg air‐dried soil)	37.87	39.67	38.70	38.10	38.33	1.137	.815
K (mg/kg air‐dried soil)	58.56	61.85	58.36	64.87	60.97	0.036^c^	.886
Ca (mg/kg air‐dried soil)	1,300	1,214	1,202	1,295	1,334	51.3	.357
Mg (mg/kg air‐dried soil)	90.57	74.09	80.37	86.32	85.53	0.411^b^	.600
Forage							
Tiller density (Tillers 0.1 m^−2^)	201.5^b^	184.8^a^	225.6^b^	225.9^b^	232.6^b^	7.42	.008

Note

Differing superscripts within rows denote statistically different means based on a Student–Newman–Keuls test (*p* < .05 except for PR where *p* < .06).

^a^ SEM applies to Box–cox transformed means, *y*’ = (*y^λ^*−1)/*λ* with *λ* = −0.5.

^b^ SEM applies to square root transformed means.

^c^ SEM applies to log_10_ transformed means.

### 3.2 Post soil compaction

#### 3.2.1 Soil

**Figure 1:**
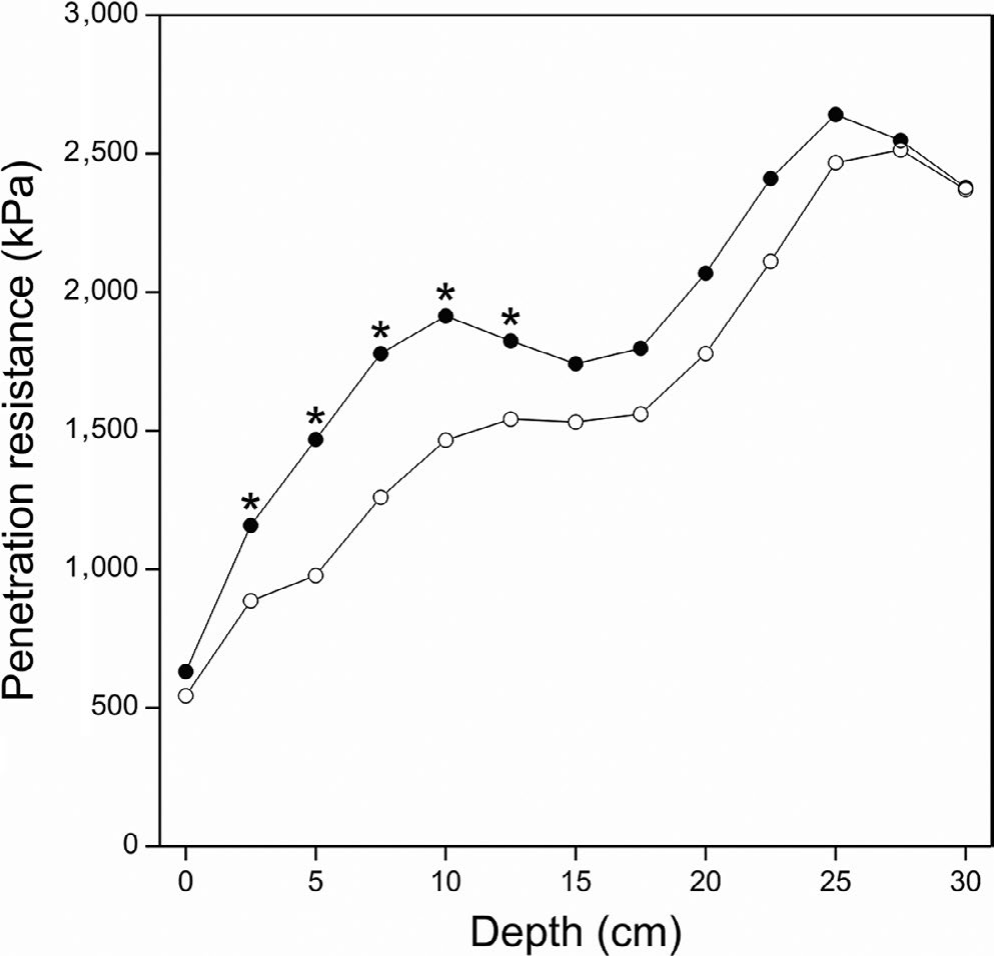
Effect of soil compaction on soil penetration resistance (0 – 30 cm) in autumn 2016. Profiles represent means across five grasses with ○ denoting non‐compacted subplots and ● subplots subjected to mechanical compaction. * denotes depths at which resistance differed (*p* < .05)

In comparison with the non‐compacted subplots, both bulk density and total penetration resistance (0 – 30 cm) were higher in compacted soils (*p* = .011 and *p* < .001, respectively) (Table 2). The PR data showed that the main effect of compaction treatment was mostly evident at a depth of 5–15 cm (Figure 1). However, while soil bulk density and total penetration resistance were both negatively affected by compaction, there was no difference between compacted and non‐compacted soil treatments in terms of water infiltration rate (*p* > .05). There were no differences in soil physical properties among the grass treatments in terms of water infiltration, bulk density, or penetration resistance (*p* > .05) (Table 2). Neither grass type nor the two soil conditions (compacted versus non‐compacted) affected soil chemical properties (Table S2), with the exception of lower NO_3_^‐^‐N under fescue than under the other grasses (*p* < .05) (Table 2). Post compaction soil mean pH was 6.27 (*SEM* ± 0.04).

**Table 2:** Effects of soil condition (SC; non‐compacted (NC) versus compacted (C)) on soil nitrate and soil physical properties: water infiltration rate (WIR), bulk density (BD), total penetration resistance 0 – 30 cm (PR) for five grasses (G), three *Festulolium* cultivars (Lp × Fm (*Lolium perenne* L. (Lp) × *Festuca mairei* Hack.), Lp × Fg (*Festuca glaucescens* Roth. (Fg)), Lp × Fp (*Festuca pratensis* Huds. (Fp)) and perennial ryegrass (Lp cv. AberBite) and tall fescue (*Festuca arundinacea* Schreb. cv. Kora) in autumn 2016

	SC	Grass (G)					*Mean*	*SEM*		*P*‐value
		Ryegrass	Fescue	Lp × Fm	Lp × Fg	Lp × Fp				
NO_3_^‐^‐*N*	NC	8.20	4.14	10.06	13.64	9.05	8.41	G	0.073^#^	.017
(mg/kg DM)	C	6.70	3.68	9.48	8.93	6.58	6.72	SC	0.044^#^	.145
	Mean	7.41^b^	3.90^a^	9.77^b^	11.03^b^	7.72^b^		G.SC	0.100^#^(17.5)	.923
								G.SC_g_	0.098^#^	
WIR	NC	3.09	2.05	3.98	3.58	3.10	3.12	G	0.217^*^	.696
(cm/h)	C	1.68	2.52	2.71	3.19	3.24	2.63	SC	0.148^*^	.506
	Mean	2.33	2.28	3.31	3.38	3.17		G.SC	0.320^*^ (17.9)	.870
								G.SC_g_	0.332^*^	
BD	NC	1.33	1.35	1.33	1.33	1.38	1.34	G	0.025	.869
(g DM cm^−3^)	C	1.41	1.42	1.42	1.37	1.38	1.40	SC	0.013	.011
	Mean	1.37	1.39	1.37	1.35	1.38		G.SC	0.033 (16.6)	.647
								G.SC_g_	0.030	
Total PR	NC	47,957	49,384	50,358	49,304	48,586	49,118	G	2055.3	.752
(kPa)	C	56,531	61,550	55,759	57,686	55,043	57,314	SC	698.3	<.001
	Mean	52,244	55,467	53,058	53,495	51,814		G.SC	2333.1 (12.5)	.311
								G.SC_g_	1561.4	

Note

Differing superscripts within rows denote statistically different means based on a Student–Newman–Keuls test (*p* < .05).

G.SC denotes *SEM* for interaction means value in parentheses indicates relevant *df*.

G.SC_g_; denotes interaction *SEM* within the same level of G with 10 *df*.

^#^ SEM applies to log_10_ transformed means.

^*^ SEM applies to square root transformed means.

#### 3.2.2 Forage

After compaction, TD numbers among the grass types did not differ significantly (*p* > .05 (Table 3) although the tall fescue, as found prior to compaction, had the lowest tiller number numerically. However, tiller densities were significantly lower (*p* < .05) in compacted soil than in non‐compacted soils (251 versus 271 tillers 0.1 m^‐2^, respectively). Tiller density in the ryegrass and tall fescue treatments was 12.7% and 3.7% lower in the compacted soil compared to the non‐compacted soil. In comparison, there was only a 0.3% reduction in tiller density for *Festulolium* cv. Lp × Fg in compacted soils, which was the lowest among the three *Festulolium* cultivars. Lp × Fm and Lp × Fp showed 9.4% and 9.8% reductions, respectively.

**Table 3:** Effects of soil condition (SC; non‐compacted (NC) versus compacted (C)) on plant tiller densities (TD) and root biomass (RB: t DM ha^‐1^) at different soil depths and total RB for five grasses (G), three *Festulolium* cultivars (Lp × Fm (*Lolium perenne* L. (Lp) × *Festuca mairei* Hack.), Lp × Fg (*Festuca glaucescens* Roth. (Fg)), Lp × Fp (*Festuca pratensis* Huds. (Fp)) and perennial ryegrass (Lp cv. AberBite) and tall fescue (*Festuca arundinacea* Schreb. cv. Kora) in autumn 2016

	SC	Grass (G)					Mean	*SEM*		*P*‐value
		Ryegrass	Fescue	Lp × Fm	Lp × Fg	Lp × Fp				
TD	NC	275.6	232.0	285.7	264.1	297.8	271.0	G	12.65	.106
(Tillers 0.1 m^−2^)	C	240.4	223.5	258.9	263.3	268.5	250.9	SC	5.17	.021
	Mean	258.0	227.7	272.3	263.7	283.1		G.SC	15.07 (14.1)	.548
								G.SC_g_	11.57	
RB	NC	9.64	7.76	10.11	10.74	7.98	9.24	G	0.695	.208
(0 – 15 cm)	C	7.28	8.70	8.06	10.34	8.78	8.63	SC	0.253	.118
	Mean	8.46	8.38	9.08	10.54	8.23		G.SC	0.802 (13.0)	.041
								G.SC_g_	0.566	
RB	NC	0.19	0.39	0.16	0.20	0.19	0.23	G	0.029	.002
(15 – 30 cm)	C	0.25	0.49	0.23	0.25	0.22	0.29	SC	0.028	.144
	Mean	0.22^a^	0.44^b^	0.19^a^	0.22^a^	0.21^a^		G.SC	0.053 (16.5)	.978
								G.SC_g_	0.063	
Total RB	NC	9.84	8.14	10.27	10.93	8.17	9.47	G	0.701	.239
(0 – 30 cm)	C	7.53	9.19	8.29	10.59	9.01	8.92	SC	0.254	.159
	Mean	8.86	8.67	9.28	10.76	8.59		G.SC	0.808 (13.0)	.042
								G.SC_g_	0.569	

Note

Differing superscripts within rows denote statistically different means based on a Student–Newman–Keuls test (*p* < .05).

G.SC denotes *SEM* for interaction means value in parentheses indicates relevant *df*.

G.SC_g_; denotes interaction *SEM* within the same level of G with 10 *df*.

Tall fescue had more roots below 15 cm (*p* < .05) than all other grasses while ryegrass and the three *Festulolium* had a similar root biomass (Table 3). Root biomass above 15 cm, below 15 cm, and overall (0 – 30 cm) showed no main effect of soil compaction (*p* > .05) (Table 3). However, there was a significant grass × soil condition interaction (*p* = .04) in terms of root biomass above 15 cm and total root biomass. This reflected a tendency (*p* > .05) for perennial ryegrass and Lp × Fm to have lower root biomass in compacted soil than in non‐compacted soil, while fescue, Lp × Fg and Lp × Fp showed relatively little difference (Table 3). *Festulolium* Lp × Fg tended (*p* > .05) to have the highest root biomass than the remaining grasses under both soil conditions.

Forage dry matter yield (DMY) at the first harvest, 9 weeks following compaction, was lower in the compacted subplots than in their non‐compacted counterparts (2.97 v 3.28 t DM ha^‐1^, respectively; *p* < .001) (Table 4). Conversely, the DMY of the compacted plots was higher (*p* < .01) than the non‐compacted at the second harvest, 15 weeks following compaction. After the second cut, there were no further effects of compaction on DMY (*p* > .05) and total DMY for all grass types accumulated over all four harvests was not significantly different between soil condition treatments *(p* > .05). Despite having lower tiller densities, the DMY from the first harvest cut of tall fescue was higher than from all other grasses *(p* < .05). Ryegrass produced a higher DMY at the second harvest cut compared to all others (*p* < .05), but in the third and fourth cuts, tall fescue had the highest yields (*p* < .05). DMY from the *Festulolium* treatments did not differ significantly (*p* > .05) from that of ryegrass in all cuts, except the second cut where they were lower (Table 4). Tall fescue produced the highest total DMY of all grass treatments (*p* < .001). Botanical composition data showed a tendency (*p* = .05) for differences among grass treatments with *Festulolium* cv. Lp × Fp plots having the numerically highest proportion of weed contamination and ryegrass and Lp × Fg the lowest. There was no effect of compaction on the unsown species yield (*p* > .05) (Table 4).

**Table 4:** Effect of soil condition (SC; non‐compacted (NC) versus compacted (C)) on dry matter yield from four cuts (17 May, 27 June, 15 August and 4 October 2016) and weed (unsown grass and broad leaved weeds) presence for five grasses (G), three *Festulolium* cultivars (Lp × Fm (*Lolium perenne* L. (Lp) × *Festuca mairei* Hack.), Lp × Fg (*Festuca glaucescens* Roth. (Fg)), Lp × Fp (*Festuca pratensis* Huds. (Fp)) and perennial ryegrass (Lp cv. AberBite) and tall fescue (*Festuca arundinacea* Schreb. cv. Kora)

	SC	Grass					Mean		*SEM*	*P*‐value
		Ryegrass	Fescue	Lp × Fm	Lp × Fg	Lp × Fp				
1st cut	NC	2.713	4.080	3.186	3.282	3.159	*3.284*	G	0.1806	.010
(t DM ha^−1^)	C	2.633	3.784	2.953	2.742	2.751	*2.973*	SC	0.0372	<.001
	Mean	2.673^a^	3.932^b^	3.070^a^	3.012^a^	2.955^a^		G.SC	0.1900 (9.7)	.143
								G.SC_g_	0.0831	
2nd cut	NC	6.127	5.594	5.199	5.353	5.146	*5.484*	G	0.1138	<.001
(t DM ha^−1^)	C	6.621	6.015	5.413	5.379	5.743	*5.834*	SC	0.0760	.009
	Mean	6.374^b^	5.804^a^	5.306^a^	5.366^a^	5.444^a^		G.SC	0.1655 (17.9)	.494
								G.SC_g_	0.1700	
3rd cut	NC	3.826	4.995	3.609	3.545	3.374	*3.870*	G	0.1464	<.001
(t DM ha^−1^)	C	3.379	5.236	3.421	3.263	3.192	*3.698*	SC	0.0601	.071
	Mean	3.602^a^	5.116^b^	3.515^a^	3.404^a^	3.283^a^		G.SC	0.1745 (14.2)	.207
								G.SC_g_	0.1343	
4th cut	NC	2.255	2.806	2.060	2.191	1.872	*2.237*	G	0.0812	<.001
(t DM ha^−1^)	C	2.125	2.784	2.067	1.920	1.912	*2.154*	SC	0.0409	.185
	Mean	2.190	2.777	2.063	2.055	1.892		G.SC	0.1038 (16.2)	.492
								G.SC_g_	0.0915	
Annual	NC	14.921	17.475	14.055	14.371	13.551	*14.875*	G	0.3631	.001
(t DM ha^−1^)	C	14.757	17.783	13.853	13.304	13.598	*14.659*	SC	0.1281	.262
	Mean	14.839^a^	17.629^b^	13.954^a^	13.838^a^	13.574^a^		G.SC	0.4158 (12.8)	.241
								G.SC_g_	0.2865	
Annual Weed	NC	44.3	340.8	425.8	142.3	378.8	*266.4*	G	71.79	.050
(kg DM ha^−1^)	C	180.6	188.5	320.9	86.9	447.7	*244.9*	SC	31.01	.635
	Mean	112.4	264.6	373.4	114.6	413.2		G.SC	86.94 (14.6)	.270
								G.SC_g_	69.35	

Note

Differing superscripts within rows denote statistically different means based on a Student–Newman–Keuls test (*p* < .05).

G.SC denotes *SEM* for interaction means value in parentheses indicates relevant *df*; G.SC_g_ denotes interaction *SEM* within the same level of G with 10 *df*.

## 4 DISCUSSION

### 4.1 Soil compaction

Given that the focus of this study was to determine whether different grasslands could be used to provide greater resilience to the effects of machinery‐derived soil compaction and to deliver ecosystem services while still maintaining agricultural productivity, it was imperative to induce a state of compacted soil in order to effectively test this hypothesis. From previous research, it has been shown that to create a measurable change in soil bulk density requires considerable changes in soil physical properties to be able to induce a statistically significant difference in soil bulk density between experimental treatments comparing contrasting forages. For example, in research showing difference in biological properties of soils due to changes in earthworm numbers (Crotty, Fychan, Scullion, Sanderson, & Marley, 2015) and soil microbial populations (Detheridge et al., 2016), significant differences in soil bulk density were not evident among treatments. Soil bulk density and penetration resistance were higher in compacted soils, (*p* = .011; *p* < .001, respectively). As shown in Figure 1, the significant differences in PR between compacted and non‐compacted plots were still found in the autumn, confirming that the effects of the soil compaction were present throughout the period of the experiment. We are therefore confident that the experimental approach did create a compacted soil to effectively test the research question being investigated. In the current study, following simulated compaction using a loam soil typical of grassland soils found in western UK, soil bulk density was confirmed to be higher within the compacted compared to the non‐compacted soils. Soil total penetration resistance in the compacted plots was also significantly higher than in the non‐compacted soils. These findings confirmed that the procedures used in the current study to simulate soil compaction by farm machinery were effective in modifying some physical properties of soil particularly in the uppermost 15 cm.

Furthermore, while only a single compaction event was employed in this study, it is more common on farmed grasslands that compaction is a repetitive process, by both livestock poaching and/or farm machinery. A repetitive compaction approach was not employed here as repeated compaction would also have potentially created irreversible effects on the plants, particularly on plant tillers and forage biomass, during the season making it impossible to decifer whether the effects observed were due to a compacted soil per se (the aim of the current study) or the effects of damage to individual plant tillers and plant material. However, it is worth noting that in such a scenario, as repeated damage from farm machinery during harvesting or poaching by livestock, different outcomes might occur and further studies are now needed to determine these effects.

It has been reported previously that machinery‐induced soil compaction leads to inferior soil physical properties, such as reduced soil porosity, hydraulic conductivity, and increased bulk density (Junior, 2003; Kozlowski, 1999; Steffens, Kölbl, Totsche, & Kögel‐Knabner, 2008; Willatt & Pullar, 1984). Gupta, Sharma, and DeFranchi (1989) and Singh, Salaria, and Kaul (2015) considered that increased soil bulk density might influence soil hydraulic conductivity and penetration resistance. In the spring, prior to soil compaction and the full commencement of the growing season, the perennial ryegrass plots showed significantly lower total PR compared to the *Festulolium* cultivars Lp × Fm and Lp × Fg. However, at the second PR measurement in the autumn of the same year, this effect had diminished, with the penetration resistance of the ryegrass treatments being equivalent to that under the other grasses. Thus, the initial PR advantage prior to soil compaction found with ryegrass compared to *Festulolium* failed to persist as the growing season progressed. Coupled with this, the current study did not find any effects of soil compaction or grass type on water infiltration rates. In previous studies by Gregory et al. (2010), comparing similar (and in some cases the same) grass cultivars, it was shown that the grass species altered the saturated hydraulic conductivity of the soil capillary matrix. Therefore, given the importance of the rates of soil drying on soil strength (Rondinelli et al., 2015), as differences in water uptake by roots was not measured during the current study, further studies are now needed to include these parameters and for the findings presented here to be evaluated across a range of different soil types to fully understand the plant genotype responses to differing soil strength.

While Mada, Ibrahim, and Hussaini (2013) concluded that compaction may alter soil physical properties, Regelink et al. (2015) linked the impact from compaction that induced poor soil physical properties (aggregate formation and porosity) with changes in soil chemical properties. Impacts of compaction on grasslands can be found: i) within the soil chemical profile and ii) the plant nutritional alignment. Arvidsson (1999) reported there to be lower phosphorus and potassium concentrations in compacted soils which had increased bulk density. Steffens et al. (2008) found intensive grazing and high stock rates can lead to higher soil bulk density and compacted soils resulting in changes in the soil chemical parameters, including soil total N and sulfur with changes persistent for five years. In contrast, findings related to the soil chemical measurements (nitrate and ammonium‐N, P, K, Ca, Mg) were not affected by compaction in the current study. However, the soil cores used here for the chemical analysis were collected 33 weeks following the compaction event and it is possible that some early impacts of compaction on the soil chemistry were undetected as a result of this time period in the current study. N uptake ability and K and Ca concentrations in forages have been found to be reduced in compacted soils compared to non‐compacted (Kuht & Reintam, 2004). Compaction increases soil bulk density which is negatively correlated with forage N, P, and K concentrations (Reintam, Kuht, Loogus, Nugis, & Trükmann, 2005). The changes in soil bulk density in the current study may have reduced nutrient uptake by grasses under soil compaction and impacted on their forage chemical composition. However, forage mineral composition was outside the scope of the current study and further work is needed to investigate these potential implications.

### 4.2 Agricultural productivity

Soil compaction impairs crop production (Gysi, 2001; McKenzie, 2010). This occurs due to suboptimal root growth following soil compaction (Nadian, Smith, Alston, & Murray, 1997) and reduced root penetration (Singh et al., 2015; Unger & Kaspar, 1994). The impacts of soil compaction on forage may be due to a reduced plant nutrient uptake and reduced root biomass at depth, the latter being a known attribute in certain *Festulolium* genotypes with improved drought resistance (Durand et al., 2007; Humphreys et al., 2018) and so these below‐ground responses were a key focus of the current study. Previous studies have shown Lp × Fm and Lp × Fg to have a higher root biomass in comparison with the same perennial ryegrass (cv Aberbite) as used in the current study when kept under similar environmental conditions and compared using more detailed root imaging to study root ontogeny (Humphreys et al., 2018). In the current study, root biomass in compacted soils showed a tendency for Festulolium cv. Lp × Fg to have a higher root biomass than the ryegrass at 0 – 15 cm depth. The root biomass results herein indicate potential for Festulolium cv. Lp × Fg to produce quantitatively more root irrespective of whether or not soils had been compacted. The perennial ryegrass cv. AberBite lost considerable root biomass (23.5%) following compaction, whereas Festulolium cv. Lp × Fg retained a proportionally higher root biomass with only 3.2% lost. In compacted soils, it can be concluded that Lp × Fg performed well in terms of below‐ground growth when compared to a UK National‐Listed perennial ryegrass control.

At the first harvest, nine weeks post compaction, a significant reduction in grass DMY was observed for compacted compared to non‐compacted soils confirming the known impact of soil compaction on forage yield, as explained by Douglas (1994). Following another six weeks, at the second harvest cut, the reverse was found with the grasses growing on the compacted plots having higher DMYs than those on the non‐compacted soils. This result was also encountered in a previous study (Douglas, 1997) using mechanically compacted and non‐compacted plots under UK conditions. In the latter, the difference in yield was explained as an effect of weather parameters. However, in the current study, the impact of compaction appeared transitory since total yield over the growing season was comparable for both compacted and non‐compacted soils across all grasses. In the current experiment, a cold spring was encountered that initially prevented significant foliar growth by all the grasses, and this might accord with the same conclusions described by Douglas (1997).

Furthermore, grasses in compacted soils had lower plant tiller densities compared with non‐compacted soils but there were no differences among the grasses. This finding was not as expected from the pre‐compaction data (showing higher tiller density for the *Festuloliums* compared to fescue, with perennial ryegrass intermediate) but is in agreement with other studies (Atwell, 1990; Nie, Ward, & Michael, 2001; Harkess, 1970, respectively). The lower tiller density of the fescue compared to the other grasses may explain why fescue had a lower water infiltration rate prior to compaction compared to the other grasses. However, despite having lower tiller numbers prior to compaction, the tall fescue had a higher overall DMY compared to the other grasses. This findings may be explained by several factors. Firstly, nitrogen is the most influential nutrient on plant growth and productivity (Leghari et al., 2016) and the significantly lower NO_3_^‐^‐N concentration observed in soil in which tall fescue was grown soil may indicate higher N uptake reflecting its higher yield. In addition, fescue is known to have greater leaf area (Marks & Clay, 2007), tall canopies (Raeside, Friend, Behrendt, Lawson, & Clark, 2012) and shows resilience to stress (Carrow & Duncan, 2003; Humphreys et al., 2006) which may also be a part of its dry matter yield potential.

However, it is worth remembering that despite the higher total yield potential of tall fescue, as shown in the current study, it is not used extensively in UK ruminant systems due to its lower forage quality compared to ryegrass (Østrem, Volden, Steinshamn, & Volden, 2015) and lower voluntary intake due to poor palatability (Cougnon, Baert, Van Waes, & Reheul, 2014). Other than the tall fescue, the national‐listed grass varieties and cultivars used in the current study have been evaluated for their DM yields and forage quality and have been found to be equivalent to NL UK perennial ryegrass varieties (Humphreys et al., 2014).

When considering total silage productivity on commercial farms, the first and second harvests (which for the UK typically occur in May and June) are expected to capture a significant part of the annual forage yield, and provide for livestock use, feed of the highest possible forage quality. It follows that evidence of soil compaction having a negative effect on grass production at these stages of the growth cycle will have a significant impact on silage production. In the current study, the negative effects of soil compaction on forage yields were evident at the first harvest, in May, nine weeks post compaction. Subsequently, following a further six weeks into the growing season, at the second harvest cut, crop growth by the grasses on the compacted soils had recovered to an extent that they exceeded the yields achieved by grasses growing on the non‐compacted soils.

Can novel grasslands provide environment‐enhancing ecosystem services while maintaining agricultural productivity?

Among all the grasses investigated, *Festulolium* Lp × Fg, reflected as a tendency for a higher root biomass than ryegrass at 0–15 cm, was the least affected following onset of soil compaction.. In the current study, root frequencies were scored only within the top 30 cm soil layer, with greater than 95 percent of the root biomass found in the uppermost 15 cm of soil. Heavy farm machinery may impact to a depth of 60 cm (McKenzie, 2010), but the most significant impact still resides within the uppermost 10 cm soil horizon (Flowers & Lal, 1998; Mkomwa, Kaumbutho, & Makungu, 2015). This is in agreement with the current study which found that the effect of compaction was mostly evident in the 5 ‐ 15cm depth of soil (Figure 1). The root biomass shown for Lp × Fg in compacted soil may indicate its potential to penetrate and be resilient to damaged soils, including those on farms compacted by animal hooves within soil depths from 5 to 20 cm (Ziyaee & Roshani, 2012).

In considering the future sustainability of grasslands, the introduction of resilient and sustainable grass varieties capable of maintaining yields equivalent to ryegrass is a major plant breeding objective and needed to support future livestock agricultural systems. Climate change increases the risk of heavy rainfall within short time periods, leading to grassland flooding which may create and be further exacerbated by soil compaction. Grasses capable of producing large root systems in compacted soil will assist organic matter accumulation (Humphreys, 2011; Misra & Gibbons, 1996), increase soil shear strength (Ekwue, 1990), and enhance bearing capacity. This is an important goal for grasslands where opportunity to accumulate organic matter over the time is limited due to continuous forage removal for silage or by grazing livestock. The accumulation of root biomass in soils aides long‐term improvements in soil physical properties that may facilitate a better soil chemical profile, and allows opportunities for carbon sequestration (Kell, 2011). Root–soil interactions that benefit soil water infiltration, whether facilitated by *Festulolium* cultivars or by other grassland species, have potential to reduce run off and to mitigate, nutrient loss, and soil erosion (Macleod et al., 2013).

## 5 CONCLUSION

The productivity of the *Festuloliums* and ryegrass control did not differ in terms of total dry matter yield, but ryegrass showed a tendency for reduced root biomass when grown in compacted soil particularly at 0 – 15 cm soil depth compared to *Festulolium* cultivar Lp × Fg. Root biomass responses to soil compaction among the *Festulolium* cultivars were variable suggesting certain species’ combinations may have an advantage over others. Supporting evidence was found for the use of *Festulolium* root–soil interactions as aides for effective soil water infiltration *per se* in non‐compacted soils in spring. Future research should now determine whether soil compaction/genotype altered forage nutrient composition and the effects of differing soil types on the findings presented here when evaluated across different sites. Overall, the findings show that alternative grass root structures provide differing resilience to soil compaction, and root biomass production can be encouraged without negative impacts on forage productivity. Grasslands with these attributes will be essential for future UK livestock systems required to provide enhanced ecosystem services without losing yield advantages.

## CONFLICT OF INTEREST

None declared.

## Supporting information

Table S1

Table S2
