# Supplementary material for: Do agricultural grasses bred for improved root systems provide resilience to machinery‐derived soil compaction?
Source: Food Energy Secur. 2020 Jul 5;9(3):e227. doi: 10.1002/fes3.227 (PMC7507784; doi:10.1002/fes3.227)
Supplement: Supplementary file 1 — Table S1 [file FES3-9-e227-s001.docx]

**TABLE S1** Mean monthly air temperatures, total monthly rainfalls and average monthly soil temperatures at 10 cm and 30 cm depths in 2016.

| Month | Air temperature  (^o^C) | Rainfall  (mm) | Soil temperature (^o^C) | |
| --- | --- | --- | --- | --- |
|  |  |  | 10 cm | 30 cm |
| January | 9.6 | 147.0 | 5.8 | 7.8 |
| February | 8.6 | 98.6 | 4.6 | 7.1 |
| March | 10.0 | 84.6 | 5.2 | 7.4 |
| April | 11.5 | 59.8 | 8.1 | 9.6 |
| May | 16.9 | 64.4 | 12.9 | 12.9 |
| June | 18.8 | 159.2 | 16.7 | 16.2 |
| July | 18.6 | 116.6 | 16.6 | 17.6 |
| August | 19.9 | 85.2 | 16.7 | 17.8 |
| September | 18.9 | 155.6 | 15.6 | 16.9 |
| October | 14.9 | 21.6 | 9.5 | 11.8 |
| November | 10.0 | 77.2 | 6.6 | 9.5 |
| December | 10.9 | 77.4 | 6.6 | 8.2 |

Source: Met office data from Gogerddan weather station (Latitude: 52.4 N; Longitude: 4 W; Altitude: 31 m)
