# Supplementary material for: Do agricultural grasses bred for improved root systems provide resilience to machinery‐derived soil compaction?
Source: Food Energy Secur. 2020 Jul 5;9(3):e227. doi: 10.1002/fes3.227 (PMC7507784; doi:10.1002/fes3.227)
Supplement: Supplementary file 2 — Table S2 [file FES3-9-e227-s002.docx]

**TABLE S2** Effects of soil condition (SC; non-compacted (NC) versus compacted (C)) on soil chemistry for five grasses (G), three *Festulolium* cultivars (Lp x Fm (*Lolium perenne* L. (Lp) x *Festuca mairei* Hack.), Lp x Fg (*Festuca glaucescens* Roth. (Fg)), Lp x Fp (*Festuca pratensis* Huds. (Fp)) and perennial ryegrass (Lp cv. AberBite) and tall fescue (*Festuca arundinacea* Schreb. cv. Kora) in autumn 2016.

|  | SC | Grass | | | | | *Mean* | SEM | | *P*-value |
| --- | --- | --- | --- | --- | --- | --- | --- | --- | --- | --- |
|  |  | Ryegrass | Fescue | Lp x Fm | Lp x Fg | Lp x Fp |  |  |  |  |
|  |  |  |  |  |  |  |  |  |  |  |
| NH_4_^+^-N | NC | 5.24 | 4.67 | 6.39 | 6.98 | 5.74 | *5.80* | G | 0.440 | 0.484 |
| (mg kg^-1^ DM) | C | 6.32 | 5.55 | 5.77 | 5.34 | 5.32 | *5.66* | SC | 0.308 | 0.747 |
|  | *Mean* | *5.78* | *5.11* | *6.08* | *6.16* | *5.53* |  | G.SC | 0.656 (18.0) | 0.319 |
|  |  |  |  |  |  |  |  | G.SC_g_ | 0.688 |  |
|  |  |  |  |  |  |  |  |  |  |  |
| P | NC | 33.57 | 34.43 | 35.03 | 31.40 | 32.43 | *33.37* | G | 1.17 | 0.459 |
| (mg kg^-1^ air-dried soil) | C | 35.53 | 33.47 | 34.47 | 33.47 | 32.00 | *33.80* | SC | 0.48 | 0.544 |
|  | *Mean* | *34.55* | *33.95* | *34.75* | *32.47* | *32.22* |  | G.SC | 1.40 (14.1) | 0.465 |
|  |  |  |  |  |  |  |  | G.SC_g_ | 1.07 |  |
|  |  |  |  |  |  |  |  |  |  |  |
| K | NC | 44.23 | 42.71 | 44.85 | 43.69 | 48.37 | *44.73* | G | 0.019^#^ | 0.388 |
| (mg kg^-1^ air-dried soil) | C | 48.44 | 47.11 | 43.75 | 44.83 | 50.64 | *46.89* | SC | 0.008^#^ | 0.106 |
|  | *Mean* | *46.29* | *44.86* | *44.30* | *44.26* | *49.49* |  | G.SC | 0.023^#^ (14.7) | 0.597 |
|  |  |  |  |  |  |  |  | G.SC_g_ | 0.018^#^ |  |
|  |  |  |  |  |  |  |  |  |  |  |
| Ca | NC | 1289 | 1311 | 1300 | 1334 | 1368 | *1320* | G | 49.5 | 0.915 |
| (mg kg^-1^ air-dried soil) | C | 1290 | 1306 | 1389 | 1322 | 1318 | *1325* | SC | 11.9 | 0.774 |
|  | *Mean* | *1290* | *1309* | *1345* | *1328* | *1343* |  | G.SC | 52.9 (10.3) | 0.199 |
|  |  |  |  |  |  |  |  | G.SC_g_ | 26.7 |  |
|  |  |  |  |  |  |  |  |  |  |  |
| Mg | NC | 107.65 | 98.18 | 101.34 | 115.50 | 110.23 | *106.40* | G | 0.048^#^ | 0.910 |
| (mg kg^-1^ air-dried soil) | C | 108.30 | 98.00 | 113.96 | 108.28 | 113.63 | *108.27* | SC | 0.007^#^ | 0.462 |
|  | *Mean* | *107.97* | *98.09* | *107.46* | *111.83* | *111.92* |  | G.SC | 0.049^#^ (8.9) | 0.231 |
|  |  |  |  |  |  |  |  | G.SC_g_ | 0.016^#^ |  |
|  |  |  |  |  |  |  |  |  |  |  |

^#^; SEM applies to log_10_ transformed means.

G.SC denotes SEM for interaction means value in parentheses indicates relevant d.f.

G.SC_g_; denotes interaction SEM within the same level of G with 10 d.f.
